# Aerobic Endurance Training Modulates Cardiac Renin‐Angiotensin System Peptides and Matrix Metalloproteinase Activity During Chronic Stress

**DOI:** 10.1111/jcmm.71218

**Published:** 2026-08-13

**Authors:** Vinicius Guzzoni, Fabiana Gomes Ferreira, Lívia Bruni de Souza, Rebecca Salomão, Nicholas Kesler, Rodrigo Yokota, Nilsa Regina Damaceno‐Rodrigues, Elia Garcia Caldini, Fernanda Klein Marcondes, Rita de Cássia Marqueti, Steven T. Haller, David J. Kennedy, Lauren G. Koch, Dulce Elena Casarini, Tatiana Sousa Cunha

**Affiliations:** ^1^ Department of Medicine, School of Medicine Federal University of São Paulo (UNIFESP) São Paulo Brazil; ^2^ Laboratory of Molecular Analyses, Faculdade de Ciências e Tecnologia em Saúde Universidade de Brasília (UnB) Distrito Federal Brazil; ^3^ Department of Physiology and Pharmacology The University of Toledo College of Medicine and Life Sciences Toledo Ohio USA; ^4^ Department of Medicine, Discipline Nephrology Universidade Federal de São Paulo (UNIFESP/EPM) São Paulo Brazil; ^5^ Laboratory of Cellular Biology (LIM 59), School of Medicine University of Sao Paulo (USP) São Paulo Brazil; ^6^ Department of Biosciences, Piracicaba Dental School University of Campinas‐UNICAMP Piracicaba São Paulo Brazil; ^7^ Department of Medicine The University of Toledo College of Medicine and Life Sciences Toledo Ohio USA; ^8^ Department of Science and Technology Institute of Science and Technology, Federal University of São Paulo (UNIFESP) São José dos Campos Brazil

**Keywords:** aerobic endurance training, angiotensin, cardiac remodelling, metalloproteinases, stress

## Abstract

To investigate the involvement of metalloproteinases (MMPs) in the context of endurance training and their interactions with renin‐angiotensin signalling (RAS) in stress‐induced pathophysiological responses. Trained rats were submitted to 8‐weeks of endurance treadmill training with chronic mild stress (CMS), TS rats, and without CMS (T). Non‐trained rats (NTS and NT groups) were submitted to identical procedures except for training. NTS and TS groups were submitted to a CMS protocol for 3 weeks. Elevated corticosterone levels indicate an initial triggering factor that mediates the downstream intracellular signalling networks related to the MMPs and RAS activation after CMS. Endurance training confers cardioprotection against the stress response by lowering the Ang 3–7 levels, whereas increased activity of MMPs was observed in TS rats. Chronic stress disrupts the cardiac function, MMPs, and RAS activation by increasing the cardiac activity of the MMP‐2 activity and Ang 1–9 levels with concomitant reduction of cardiac and blood levels of Ang 1–7. Stress also reduced circulating levels of Ang 1–9 and Ang 1–5, Ang 1–7 metabolites, along with increased Ang III (2–8). Aerobic endurance training is a potential exercise‐based strategy that counteracts the stress‐related disturbances in the MMPs and RAS signalling pathways.

## 1 Introduction

The demands of modern life, driven by rapid technological advancements, are central features of contemporary civilization. Emotional stress, overwork, financial challenges, and difficulties in adapting to these swift changes are increasingly recognized as contributors to both acute and chronic diseases [1, 2]. Among these, chronic stress has emerged as a critical risk factor, strongly associated with psychological disorders, including clinical depression, and capable of disrupting essential physiological processes, particularly those linked to metabolic and cardiovascular health [3].

Despite inter‐strain and species‐specific behavioural variations in rodent models [4, 5] stress‐induced physiological disruptions are well‐documented contributors to compromised cardiovascular health, including myocardial dysfunction, membrane destabilization, and prolonged ventricular depolarization and repolarization [6, 7]. Chronic mild stress (CMS), recognized as a reliable rodent model for inducing chronic stress and simulating depression‐like states [4], further exemplifies these effects. Studies conducted by our group have demonstrated that CMS leads to cardiac microvascular remodelling [8] and cardiometabolic complications [9], highlighting the significant cardiovascular impact of chronic stress. Conversely, aerobic training appears to mitigate some of these adverse effects, and stressed rats subjected to exercise exhibited reduced cardiac serotonin levels, circulating adrenaline, and lipid profile. Furthermore, aerobic endurance training significantly affects the renin‐angiotensin system (RAS) by reducing the stress‐induced higher circulating and cardiac angiotensin‐converting enzymes (ACE)/ACE2 ratio, highlighting the cardioprotective potential role of physical activity [9]. This observation aligns with robust evidence supporting the benefits of physical fitness in alleviating stress‐related disorders [10, 11].

While myocardial matrix metalloproteinases (MMP‐2 and MMP‐9) are important contributors to cardiovascular remodelling, particularly in response to various forms of exercise [12, 13, 14, 15], their involvement in stress‐related disorders remains underexplored. Emerging evidence suggests a potential interplay between the RAS and MMP activity in the context of cardiovascular remodelling. The classical RAS axis, often upregulated by chronic stress [8, 16, 17], has been shown to induce maladaptive extracellular matrix (ECM) remodelling through pathways involving oxidative stress and inflammation [18, 19], which are known activators of MMPs [20]. Conversely, the activation of the counter‐regulatory RAS axis, characterized by angiotensin‐(1–7) and its receptor MAS, may exert protective effects by attenuating MMP activation and limiting extracellular matrix degradation. This dual influence of the RAS on MMP activity could offer new insights into the mechanisms underlying stress‐induced microvascular remodelling and cardiometabolic complications, as observed in chronic mild stress models [8, 9, 21, 22, 23].

Given this background, our study aims to investigate the effects of aerobic endurance training and chronic stress on systemic RAS responses and cardiac remodelling. We hypothesize that endurance training not only improves systemic and cardiac RAS profiles but also modulates MMP activity by reducing Ang II levels and MMP‐9 activity while concurrently increasing Ang 1–7 levels and MMP‐2 activity.

## 2 Materials and Methods

### 2.1 Animal Care and Use

This study included male Wistar rats at 8 weeks‐old divided into four groups (*n* = 8/group): non‐trained (NT), endurance trained (T), non‐trained submitted to chronic stress (NTS), and endurance trained submitted to chronic stress (TS). Rats were maintained in a controlled room (22°C ± 2°C, 12:12‐h light–dark cycle) as described previously [9]. All procedures were approved by the Ethics in Research Committee of the Federal University of Sao Paulo (UNIFESP, 8842170621) and in accordance with the ARRIVE (Animal Research: Reporting of In Vivo Experiments) guidelines [24].

### 2.2 Study Protocol

**FIGURE 1:**
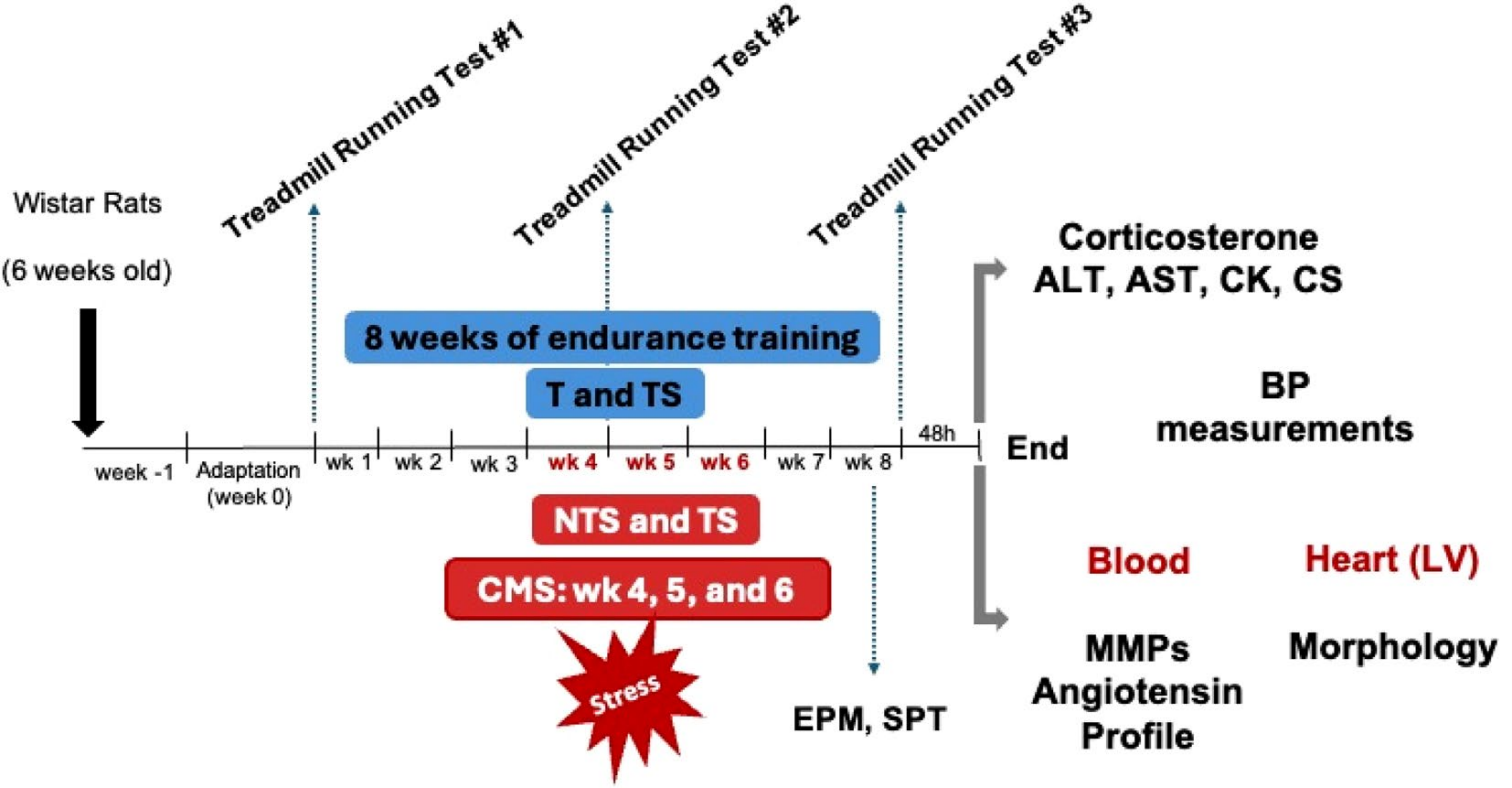
Schematic representation of the experimental design illustrating the timeline of treadmill adaptation, treadmill running tests, experimental interventions (endurance training and chronic mild stress), behavioural assessments, and biochemical analyses. ALT, Alanine aminotransferase; AST, aspartate aminotransferase; BP, blood pressure; CK, creatine kinase; CMS, chronic mild stress; CS, citrate synthase; EPM, elevated plus maze test; LV, left ventricle; MMPs, matrix metalloproteinases; NT, non‐trained rats; NTS, non‐trained rats exposed to chronic stress; SPT, sucrose preference test; T, rats subjected to endurance training; TS, rats subjected to both endurance training and chronic stress.

All animals were subjected to an initial maximal treadmill running test (Test 1) to determine baseline exercise capacity and to adjust the intensity (velocity) of the endurance training, as previously described [25]. Animals were then randomized into experimental groups based on Test 1 performance using a stratified approach to ensure comparable baseline exercise capacity across groups and to avoid allocation bias. Two additional tests were subsequently performed to further refine training velocity and assess performance levels (Tests 2 and 3) [26]. Rats assigned to the aerobic endurance training protocol (T and TS) underwent 60‐min exercise sessions on a treadmill using an intensity of 50%–70% of the maximum treadmill speed. Rats began the treadmill training at 4 pm, aligning closely with the onset of the rats' active (dark) phase to reduce potential circadian disruption and minimize stress [9, 27]. In contrast, non‐trained animals (NT and NTS groups) were placed on the treadmill once per week at a constant speed of 5 m/min for 10 min to control for handling and environmental exposure [28]. After three weeks of aerobic endurance training, rats in the TS and NTS groups were exposed to the chronic mild stress (CMS) protocol, corresponding to experimental weeks 4, 5, and 6 (Figure 1). A variety of stressors, including restraint stress, continuous overnight lighting, cage tilting, damp bedding, and reversal of the light–dark cycle, were applied over a three‐week period, twice daily, five days per week, following the protocol detailed in our previous study [9]. Animals were euthanized 60 h after the final training session by decapitation without prior anaesthesia to avoid potential increases in corticosterone associated with anaesthetic agents [29]. Following the final training session conducted on Friday afternoon, animals were euthanized on the subsequent Monday morning, corresponding to approximately 60 h after the last treadmill exercise session. Thus, all tissues were collected at least 48 h after the final bout of exercise. This interval was selected to evaluate the cumulative adaptations to treadmill training while minimizing potential confounding effects associated with the acute exercise session. This approach is consistent with previous studies [30, 31, 32, 33, 34]. Blood and heart samples were collected and stored at −80°C for subsequent biochemical analyses, and body weight (BW) was monitored throughout the entire experimental period. Biochemical measurements were performed using biological replicates, with each sample representing an individual animal. Assays were conducted in technical duplicates to ensure measurement reliability. To minimize potential bias, all biochemical measurements and histological quantifications were conducted by investigators blinded to group allocation. Samples were anonymized and coded before analysis, and decoding was performed only after completion of data collection and initial analysis.

### 2.3 Plasma Corticosterone Analysis

Corticosterone levels in plasma were measured using an enzymatic colorimetric assay kit (Enzo Life Science, Ann Arbor, MI, USA), which has a sensitivity of 0.027 ng/mL. The intra‐assay and inter‐assay coefficients of variation were 7.7% and 9.7%, respectively [8, 9].

#### 2.3.1 Tail‐Cuff Plethysmography

Forty‐eight hours after the final session of aerobic endurance training (groups T and TS), all animals underwent non‐invasive blood pressure assessment using tail‐cuff plethysmography (BP‐2000 Blood Pressure Analysis System, Visitech Systems Inc., North Carolina, USA). Systolic blood pressure (SBP), mean blood pressure (MBP), and heart rate (HR) were recorded. During each assessment, animals were placed on a heated platform (36°C) and gently restrained in a size‐adjusted holder to minimize movement and stress. A cuff equipped with a pneumatic pulse sensor was positioned near the base of the tail to allow occlusion and detection of blood flow, while the tail was secured with adhesive tape to further reduce motion artifacts. Prior to data acquisition, approximately 10 acclimation (‘warm‐up’) cycles were performed to ensure stable and reliable pulse detection. Subsequently, 30 consecutive measurements were obtained at 10‐s intervals. Up to two animals were assessed simultaneously, following the manufacturer's recommended settings [35].

### 2.4 Determination of MMP Activity by Zymography

To access the metalloprotease activity in the extracted cardiac tissue (left ventricle) and blood, zymography was performed as described previously [36]. Muscle samples were homogenized in extraction buffer containing [10 mM acid cacodylic, pH 5.0; 0.15 M NaCl; 1 μM ZnCl_2_; 20 mM CaCl_2_; 1.5 mM NaN_3_ and 0.01% Triton X‐100] (25 mg of buffer for each mg of tissue). The extraction buffer was collected by centrifugation (30 min, 4°C at 13,000 rpm). For electrophoresis, 10 μg of protein was mixed with 10 μL of a sample buffer solution without β‐mercaptoethanol. Samples were loaded onto a 10% polyacrylamide gel containing SDS and gelatin at final concentration of 1 mg/mL. To identify latent, intermediate, and active MMPs isoforms, 5 μL of the pre‐stained molecular mass marker (SM0671—Fermentas) were added to the gel. After the electrophoresis, the gel was washed twice for 20 min in a 2.5% Triton X‐100 solution to remove SDS. The gel was incubated in the substrate buffer (50 mM Tris–HCl pH 8.0, 5 mM CaCl_2_, 0.02% NaN_3_, and 10 mM ZnCl_2_) at 37°C for 18 h. The gel was stained with Coomassie Blue Brilliant R‐250 (Bio‐Rad) and destained with acid acetic, methanol and water (1:4:5) to visualize enzymatic activity. Gels were run under standardized conditions for approximately 5 h and 30 min. Densitometric analysis was performed using the InGenius imaging system with GeneSys for image acquisition and GeneTools for band quantification (Syngene, Frederick, MD, USA).

### 2.5 HPLC Analysis of Angiotensin Peptides

Angiotensin levels in the left ventricle (LV) and plasma were measured. LV was homogenized in 30 mM phosphate buffer (50 mg/0.5 mL of buffer), pH 7.4. Samples were processed for 40 s in a precooled 2 mL microcentrifuge tube containing stainless steel beads. A protease inhibitor cocktail (1 tablet/10 mL of extraction solution, Complete Mini Protease Inhibitor, Roche, USA) was added at a ratio of 10 mL per gram of tissue and used in the homogenization step. The homogenates were centrifuged at 1520 g for 10 min, and the supernatant was collected for angiotensin quantification. Solid‐phase extraction was carried out with the addition of the internal standard Sar1‐Leu8 Ang II, using Sep‐Pak C18 3 cc columns (Waters, USA). Extraction controls were performed using a tenfold concentration of the target peptides: Ang I, Ang II, Ang 1–7, Ang 1–5, Ang 1–9, Ang III (2–8), and Ang 3–7 (Sigma, USA). Peptides were separated using a Luna C18 reverse‐phase column (100 × 2.0 mm, 3 μm, Phenomenex, USA) with a linear gradient of mobile phase A (H_2_O, ammonium acetate, and formic acid) and mobile phase B (methanol, ammonium acetate, and formic acid), run on an Agilent 1260 HPLC system (Agilent, USA). The eluate was subsequently injected into a mass spectrometer coupled to the HPLC system (AB Sciex 5500 QTRAP, Sciex, USA). Peptide detection was performed via multiple reaction monitoring (MRM), based on their specific mass‐to‐charge ratio (*m*/*z*) and retention time. Quantification was performed using a calibration curve (matrix‐based) ranging from 0.25 fmol to 200 fmol per injection. Sample concentrations were determined by comparing the peak area of target ions to the known concentrations of reference peptides analysed under identical conditions. Data were analysed using Multiquant 3.0.3 (Sciex, USA) and expressed as fmol/mg [37, 38].

### 2.6 Determination of Serum of Alanine Aminotransferase (ALT) and Aspartate Aminotransferase (AST)

Both alanine and aspartate aminotransferases (ALT, and AST, respectively) activities were measured in blood by using commercially available kits (Labtest Diagnóstica S.A., Lagoa Santa, Minas Gerais, Brazil) according to the manufacturer's instructions [38].

### 2.7 Citrate Synthase (CS) Measurement

Citrate Synthase (CS) activity was measured in soleus and gastrocnemius muscles by using a commercially available kit (Citrate Synthase Activity Assay Kit; Sigma‐Aldrich, Cat# MAK193; St Louis, Missouri, United States) according to the manufacturer's instructions.

### 2.8 Creatine Kinase (CK) Analysis

Circulating creatine kinase (CK) activity and CK‐MB isoenzyme were assessed spectrophotometrically at 37°C by using a commercial enzymatic kit (Labtest Diagnóstica S.A., Lagoa Santa, Minas Gerais, Brazil; ref.#118) according to the manufacturer's instructions.

### 2.9 Assessment of Angiotensin II Type I Receptor (AT1r) Levels

Cardiac levels of the angiotensin II type I receptor (AT1r) were assessed by using a commercial enzymatic kit (LS Bio, Catalogue No. F10770) according to the manufacturer's instructions.

### 2.10 Elevated Plus Maze Test (EPM)

Anxiety‐like behaviour in the animals was assessed using the Elevated Plus Maze (EPM) test, as previously described [39]. The EPM apparatus was constructed from painted plywood and consisted of two open arms (50 cm long × 10 cm wide) and two closed arms (50 cm long × 10 cm wide, with 40 cm high walls), extending from a central platform and elevated 50 cm above the floor. The open arms lacked side walls, featuring only a peripheral ledge (1 mm wide × 5 mm high) to prevent the rats from falling. After being transferred to the experimental room, rats were allowed a 30‐min habituation period prior to testing. Each animal was then placed in the center of the maze, facing one of the closed arms, and permitted to explore the apparatus for 5 min. The maze was cleaned with 70% ethanol between trials to eliminate olfactory cues. Behavioural scoring was performed by an observer blinded to the experimental groups, recording the time spent in open and closed arms, the number of entries into each type of arm, and the total number of arm entries. The percentage of time spent in the open arms was calculated using the formula: (100 × time in open arms/total time in all arms). The EPM analysis was based on adaptations from earlier studies. The percentage of time spent in the open arms was used as an index of anxiety‐like behaviour, while the number of entries into the closed arms and the total distance travelled by the rat were used as indicators of exploratory activity. The percentage of open arm entries (100 × open arm entries/total entries) and the total number of entries were considered mixed indices reflecting both anxiety and general locomotor activity [40].

### 2.11 Sucrose Preference Test (SPT)

The Sucrose Preference Test (SPT) was employed to evaluate anhedonia by measuring sucrose consumption at the conclusion of the experimental protocol. Following 8 h of food and water deprivation, two pre‐weighed plastic bottles—one containing water and the other a 1% sucrose solution—were placed in the animals' home cage. The rats were allowed access to both fluids for a period of 1 h, after which the bottles were reweighed to determine the volume of sucrose solution and water consumed. Sucrose preference was calculated using the formula: (100 × sucrose intake/total fluid intake) [40].

### 2.12 Cardiomyocyte Width

The heart was rapidly excised, and the left ventricle (LV) was carefully dissected for morphological analysis. LV samples were fixed in 4% paraformaldehyde prepared in phosphate‐buffered saline for 24 h. Following fixation, tissues were dehydrated through graded ethanol and subsequently embedded in paraffin for histological processing. The LV was serially sectioned at a thickness of 6 μm, with slices obtained perpendicular to the longitudinal axis of the heart from base to apex. Histological sections were examined using a Nikon Opitphot microscope. Five randomly selected fields from haematoxylin and eosin‐stained LV sections were captured at 40× magnification and analysed using ImageJ 1.45S software (Media Cybernetics, Silver Spring, MD). Seven cardiomyocytes per image (*n* ≈ 35 cells per animal) were measured. For the assessment of cardiomyocyte width, as a morphometric indicator of structural remodelling, myocytes with clearly defined lateral borders were selected. The distance between the lateral boundaries, measured perpendicular to the longitudinal axis of each cell, was recorded as cardiomyocyte width. To minimize potential orientation bias, measurements were restricted to appropriately oriented fibres with visible nuclei.

### 2.13 Data Analysis

All data were assessed for normality using the Shapiro–Wilk and Kolmogorov–Smirnov tests, and for homogeneity of variances using Bartlett's test. Group differences were evaluated using two‐way analysis of variance (ANOVA), considering training (non‐trained vs. trained) and chronic stress (absence vs. presence) as independent factors. When appropriate, Tukey's post hoc test was applied for multiple pairwise comparisons among the four experimental groups (NT, T, NTS, and TS). Tukey's test was used to control the family‐wise error rate across all pairwise comparisons within each ANOVA model, thereby reducing the risk of type I error inflation within each outcome. No global correction across different endpoints was performed, as these variables represent distinct physiological domains defined a priori. Body weight gain and exercise performance were analysed using repeated‐measures one‐way ANOVA, followed by Sidak's post hoc test. Statistical analyses were performed using GraphPad Prism 6 software. Data are presented as mean ± standard error of the mean (SEM) and statistical significance was set at *p* < 0.05.

## 3 Results

**FIGURE 2:**
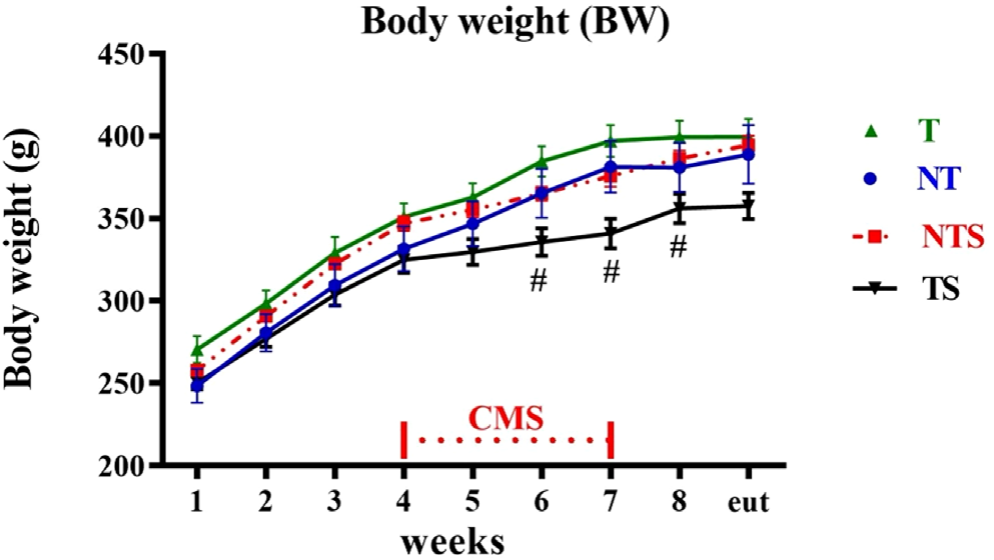
Body weight (BW) gain throughout the experimental period. Experimental groups include non‐trained rats (NT), rats subjected to endurance training (T), non‐trained rats exposed to chronic stress (NTS), and rats subjected to both endurance training and chronic stress (TS). CMS, Chronic mild stress; eut: BW measured immediately prior to euthanasia. Data are presented as means ± SEM (*n* = 8 per group). Statistical analysis was performed using repeated‐measures one‐way ANOVA or a mixed‐effects model (REML), followed by Sidak's post hoc test. Two‐way ANOVA followed by Tukey's post hoc test revealed a significant main effect of stress at week 5 (*p* = 0.01) and a significant interaction between stress and training from week 5 to week 8 (*p* < 0.03). ^#^ *p* < 0.05 vs. T group.

Body Weight (BW) was not different among the groups at the onset of the experimental protocol (week 1). All four experimental groups exhibited an increase in BW over the 8‐week aerobic endurance training period. However, the BW of stressed rats (NTS and TS groups) did not increase during the CMS protocol (week 4 vs. week 6, 7, and 8; One‐Way ANOVA followed by Sidak post hoc test). When compared to the experimental groups, TS rats exhibited reduced BW gain compared to the T group between the end of week 5 and week 7; (^#^ *p* < 0.0001; Figure 2).

**FIGURE 3:**
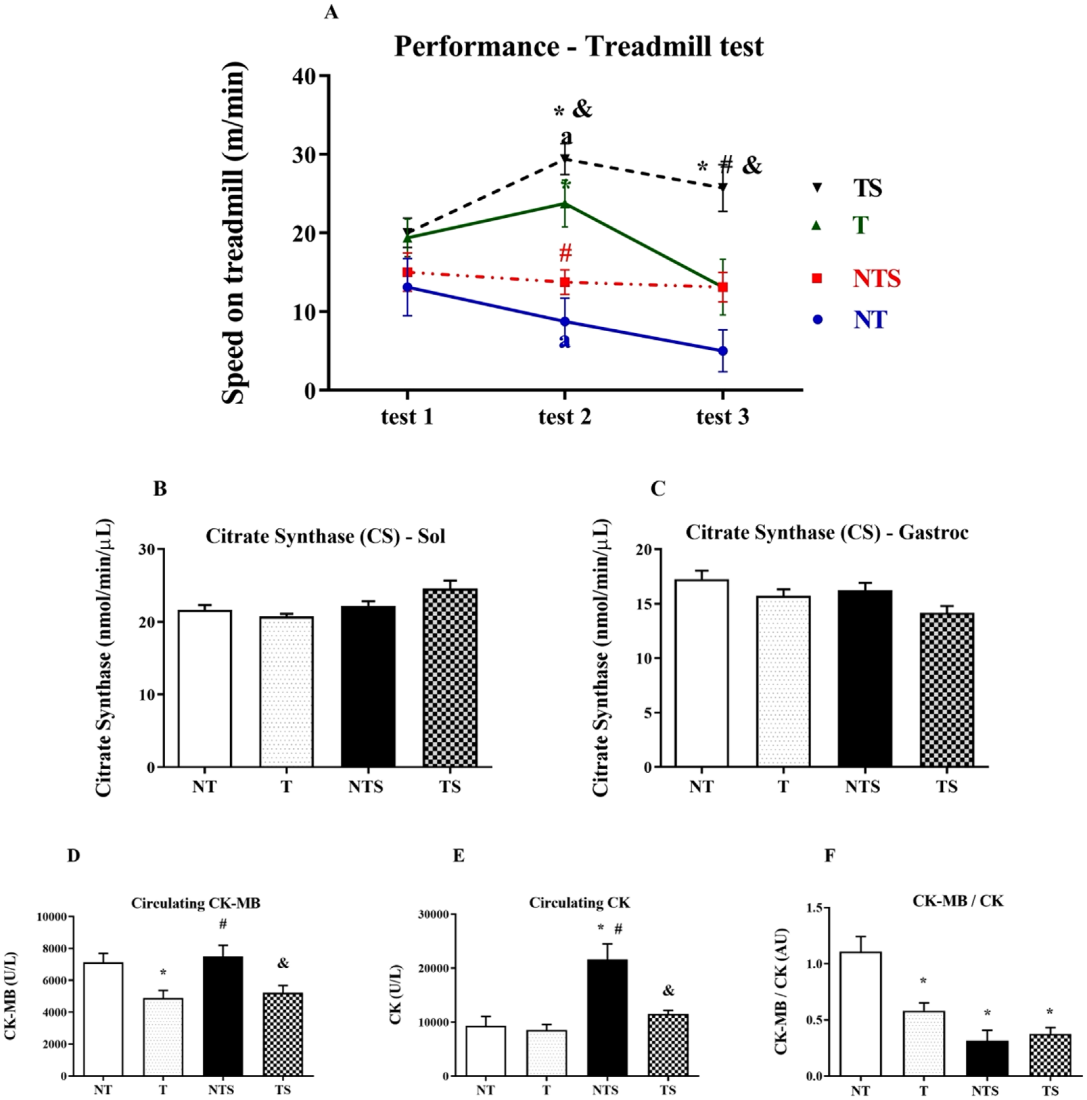
Maximal running test performance assessed at the pre‐training period (Test 1), and at weeks 3 (Test 2) and 7 (Test 3) (A); Citrate Synthase (CS) activity in the soleus (SOL) (B) and gastrocnemius (Gastroc) (C) muscles; circulating levels of creatine kinase MB isoenzyme (CK‐MB) (D), total CK (E), and CK‐MB/total CK ratio (F). Experimental groups: Non‐trained rats (NT), rats subjected to endurance training (T), non‐trained rats exposed to chronic stress (NTS), and rats subjected to both endurance training and chronic stress (TS). Values are presented as mean ± SEM (*n* = 8 per group). Statistical analysis was performed using repeated‐measures one‐way ANOVA followed by Sidak's post hoc test (a: *p* < 0.0001 vs. Test 1 within the same group). Two‐way ANOVA followed by Tukey's post hoc test revealed a significant effect of stress (*p* = 0.03) and training (*p* < 0.0001) on Test 2, and a significant effect of both stress and training (*p* = 0.001) on Test 3. *vs. NT; ^#^vs. T, and ^&^vs. NTS.

Endurance exercise performance improved in trained (T) rats subjected to CMS (repeated measures, one‐way ANOVA: ^a^vs. test 1, *p* = 0.01), whereas NT rats' performance decreased in test 2 compared to the initial test 1 (^a^vs. test 1; RM, One‐way ANOVA. *p* = 0.04; Figure 3A). The performance of TS rats was also greater than that of the NT and NTS groups (Two‐way ANOVA; main effect of stress, *p* = 0.03, and main effect of training, *p* < 0.0001). In test 2, T rats increased endurance performance more than NT (Two‐way ANOVA; main effect of training, *p* < 0.0001), whereas NTS rats showed impaired performance in comparison to the T rat group (Two‐way ANOVA; main effect of stress, *p* = 0.03). After 8 weeks of training (test 3), the TS group performed better than all the other experimental groups (Two‐way ANOVA; main effect of stress and main effect of training, *p* = 0.001; Figure 3A). No changes were observed in the CS levels of soleus and gastrocnemius muscles (Figure 3B,C). Circulating CK levels, such as isoenzyme CK‐MB (Two‐way ANOVA; main effect of training, *p* = 0.0004) and CK total (Two‐way ANOVA; interaction effect, *p* = 0.02), increased in NTS groups even though endurance training reduced the stress‐related elevated levels of CK (Figure 3D,E). Isoenzyme/CK total ratio showed reduced levels in T, NTS, and TS groups (* vs. NT, Two‐way ANOVA; interaction effect, *p* = 0.003) (Figure 3F).

**FIGURE 4:**
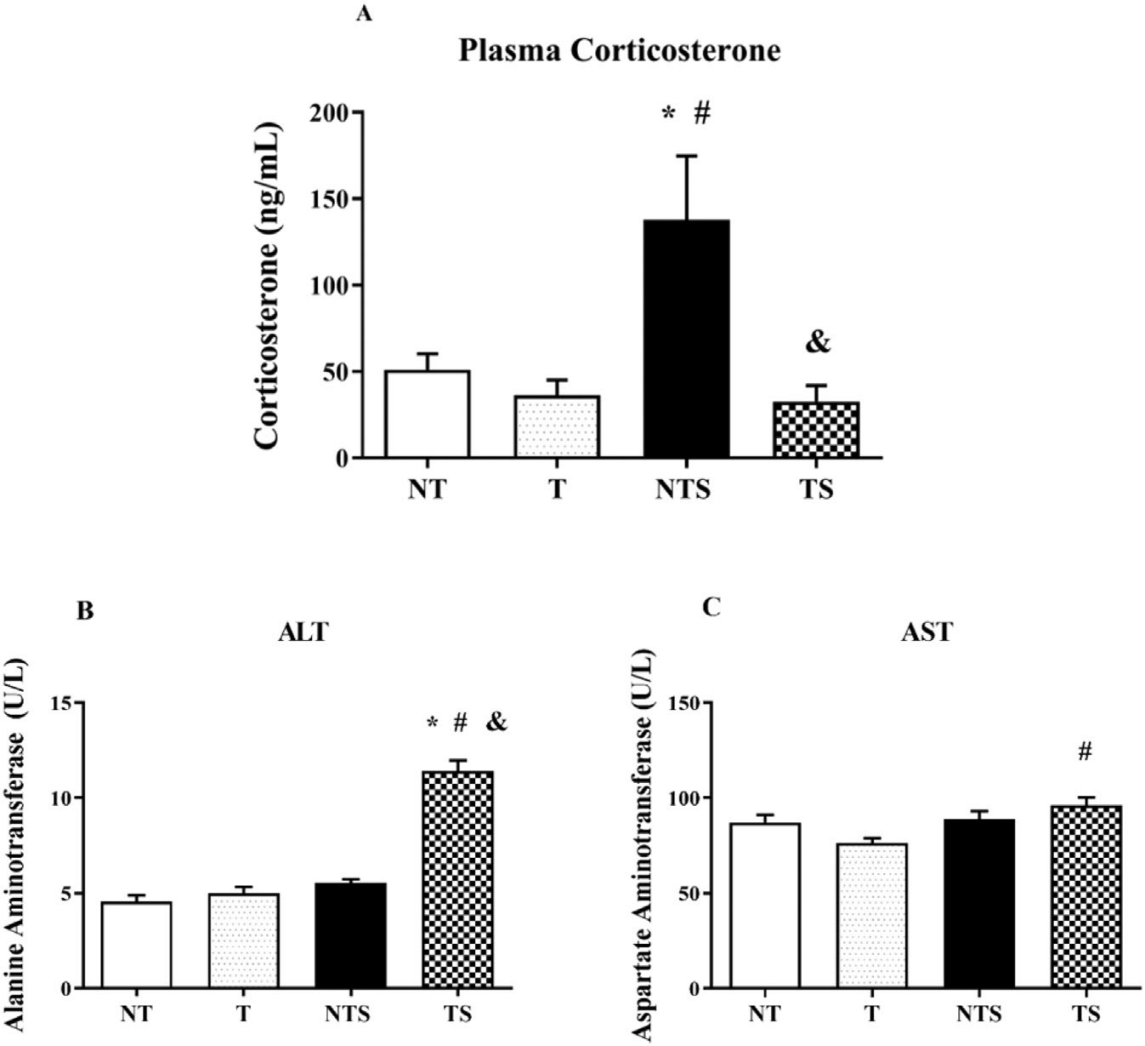
Circulating levels of corticosterone (A), alanine aminotransferase, ALT (B), and aspartate aminotransferase, AST (C). Experimental groups: Non‐trained rats (NT); rats submitted to endurance training (T); non‐trained rats submitted to chronic stress (NTS), and rats submitted to endurance training and chronic stress (TS). Data are presented as means ± SEM (*n* = 8 per group). Two‐way ANOVA followed by Tukey post hoc test revealed a significant interaction between stress and training factors (corticosterone, *p* = 0.02; ALT, *p* < 0.0001; and AST, *p* = 0.02). *vs. NT; ^#^vs. T; ^&^vs. NTS.

Blood levels of corticosterone were significantly elevated in the NTS rats compared to the NT and T groups. In contrast, aerobic training significantly reduced the stress‐associated elevated levels of circulating corticosterone in the TS group compared to the NTS group (*p* = 0.02; Figure 4A). Additionally, circulating ALT levels were significantly higher in the TS rats compared to the other groups (NT, T, and NTS; Figure 4), while AST levels were only elevated in the TS group compared to the T rats (Figure 4).

**FIGURE 5:**
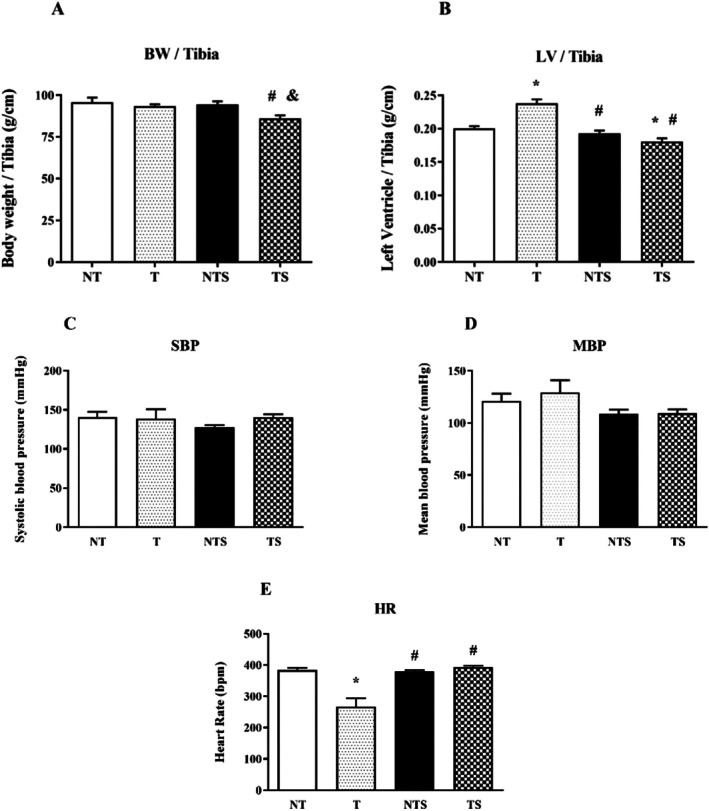
Ratios of BW to tibia length (A), and left ventricle (LV) weight to tibia length (B); blood pressure parameters including systolic blood pressure (SBP) (C), mean blood pressure (MBP) (D), and heart rate (HR) (E). Experimental groups: Non‐trained rats (NT); rats submitted to endurance training (T); non‐trained rats submitted to chronic stress (NTS), and rats submitted to endurance training and chronic stress (TS). Data are presented as mean ± SEM (*n* = 8 per group). Two‐way ANOVA followed by Tukey's post hoc test indicated a main effect of stress on BW/tibia (*p* = 0.02), a significant interaction between stress and training for LV/tibia ratio and HR (*p* < 0.0001). BW, body weight; LV, left ventricle. *vs. NT; ^#^vs. T; ^&^vs. NTS.

Body weight/tibia length ratio was reduced in TS rats (vs. T and NTS; *p* = 0.02; Figure 5A). While T rats showed an increased left ventricle weight/tibia length ratio than NT rats, a lower LV/tibia was observed in TS rats (*p* < 0.0001). Moreover, both stressed groups showed lower LV/tibia than T rats (*p* < 0.0001; Figure 5B). No changes were observed in the systolic and mean blood pressure (SBP and MBP, respectively; Figure 5C,D). The heart rate (HR) was reduced in T rats (vs. NT) even though it was significantly elevated in both stressed groups in comparison to trained rats (vs. T; *p* < 0.0001; Figure 5E).

**FIGURE 6:**
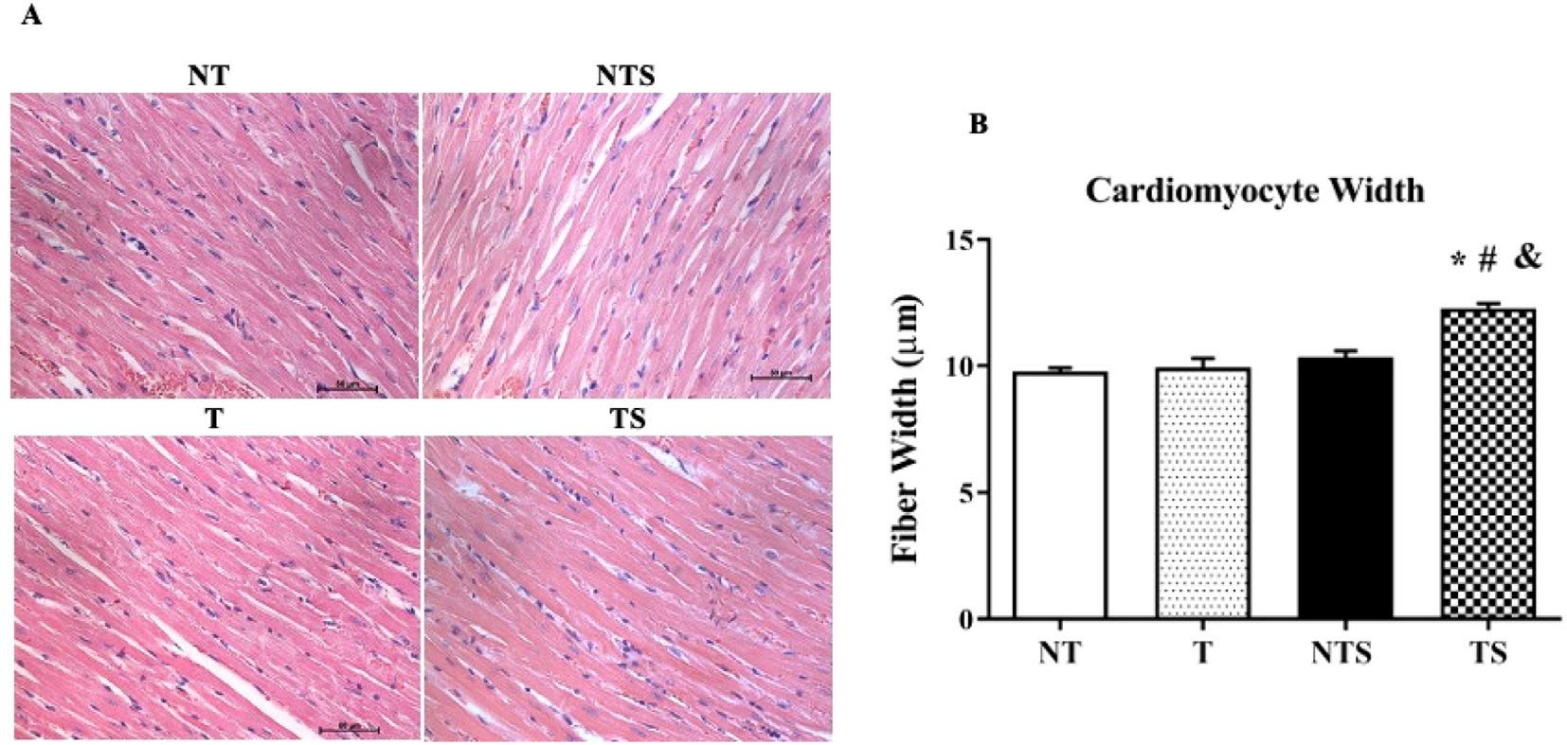
Representative images of haematoxylin and eosin (H&E) staining of left ventricle (LV) tissue at 40× magnification (A) and quantitative analysis of LV fibre width (μm) (B). Experimental groups: Non‐trained rats (NT); rats submitted to endurance training (T); non‐trained rats submitted to chronic stress (NTS), and rats submitted to endurance training and chronic stress (TS). Data are presented as means ± SEM (*n* = 8 per group). Two‐way ANOVA test followed by Tukey post hoc test revealed a significant interaction between stress and training factors (*p* = 0.001). *vs. NT; ^#^vs. T; ^&^vs. NTS.

As a morphometric indicator of structural remodelling, the cardiomyocyte width was evaluated in the LV tissue sections. Cardiomyocyte width was significantly greater in the TS group compared to the NT, T, and NTS groups (*p* = 0.001; Figure 6A,B).

**FIGURE 7:**
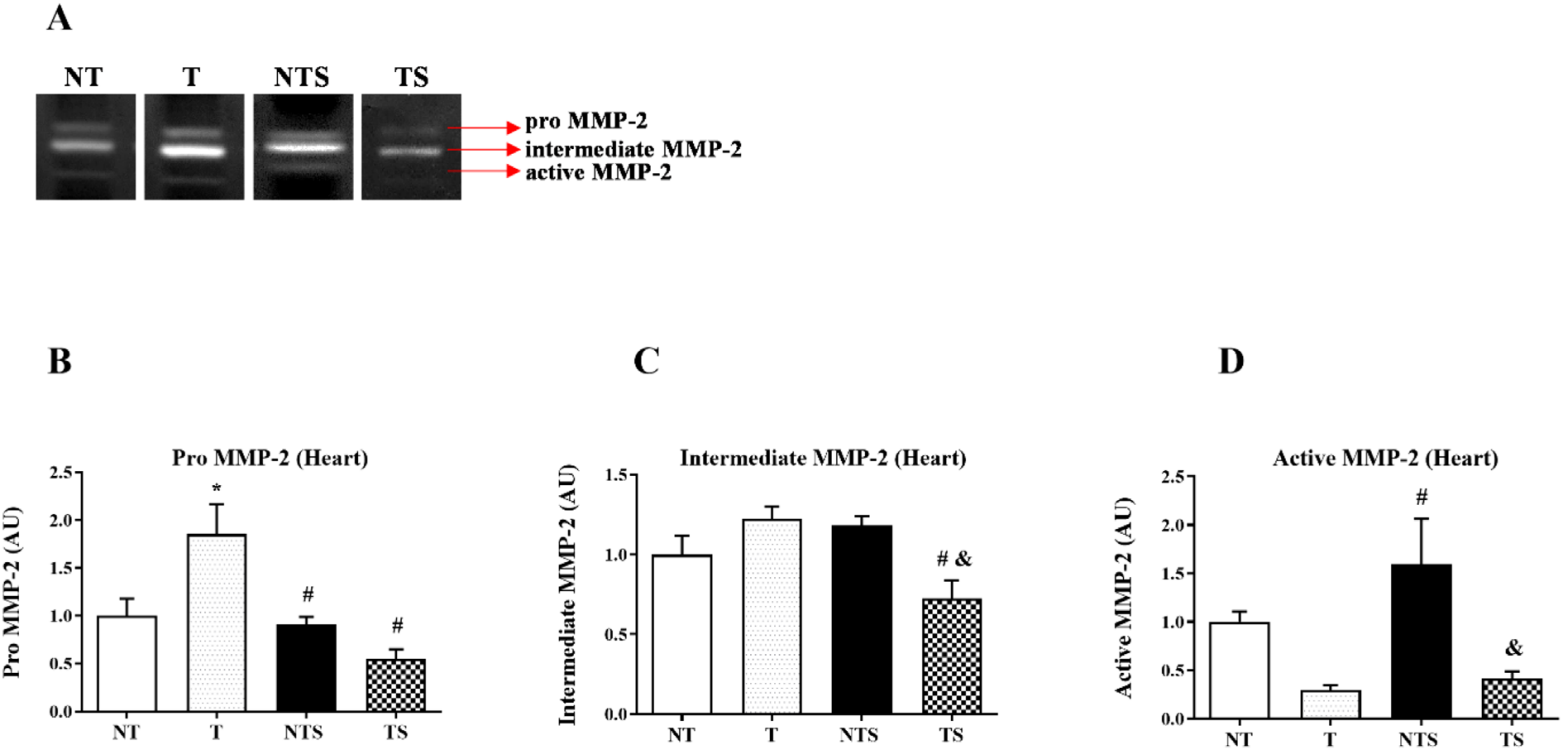
Representative gel bands showing the pro, intermediate, and active isoforms of cardiac MMP‐2 (A) with densitometry analysis of the pro (B) intermediate (C), and active (D) isoforms. Experimental groups: Non‐trained rats (NT); endurance‐trained rats (T); non‐trained rats submitted to chronic stress (NTS), and endurance‐trained rats subjected to chronic stress (TS). Data are presented as means ± SEM (*n* = 8 per group). Two‐way ANOVA followed by Tukey post hoc test revealed a significant interaction between stress and training for pro MMP‐2 (*p* = 0.02) and pro MMP‐9 (*p* = 0.01), a main effect of stress on active MMP‐2 (*p* = 0.0003) and active MMP‐9 (*p* = 0.009), and a main effect of training on active MMP‐2 (*p* = 0.0005) and active MMP‐9 (*p* = 0.001). *vs. NT; ^#^vs. T; ^&^vs. NTS.

The pro‐enzymatic form of cardiac MMP‐2 increased by 85% in the hearts of T rats compared to the NT group. In contrast, both stressed groups exhibited significantly lower cardiac levels of pro MMP‐2 compared to the T group, with NTS and TS rats showing reductions of 95% and 131%, respectively (*p* = 0.006; Figure 7A,B). The intermediate enzymatic form of cardiac MMP‐2 decreased by 50% in TS rats compared to the T group and by 46% compared to the NTS group (*p* = 0.001; Figure 7A,C). Additionally, the active enzymatic form of cardiac MMP‐2 increased by 130% in NTS rats, whereas it decreased by 118% in TS rats when compared to the NTS group (*p* = 0.002; Figure 7A,D).

**FIGURE 8:**
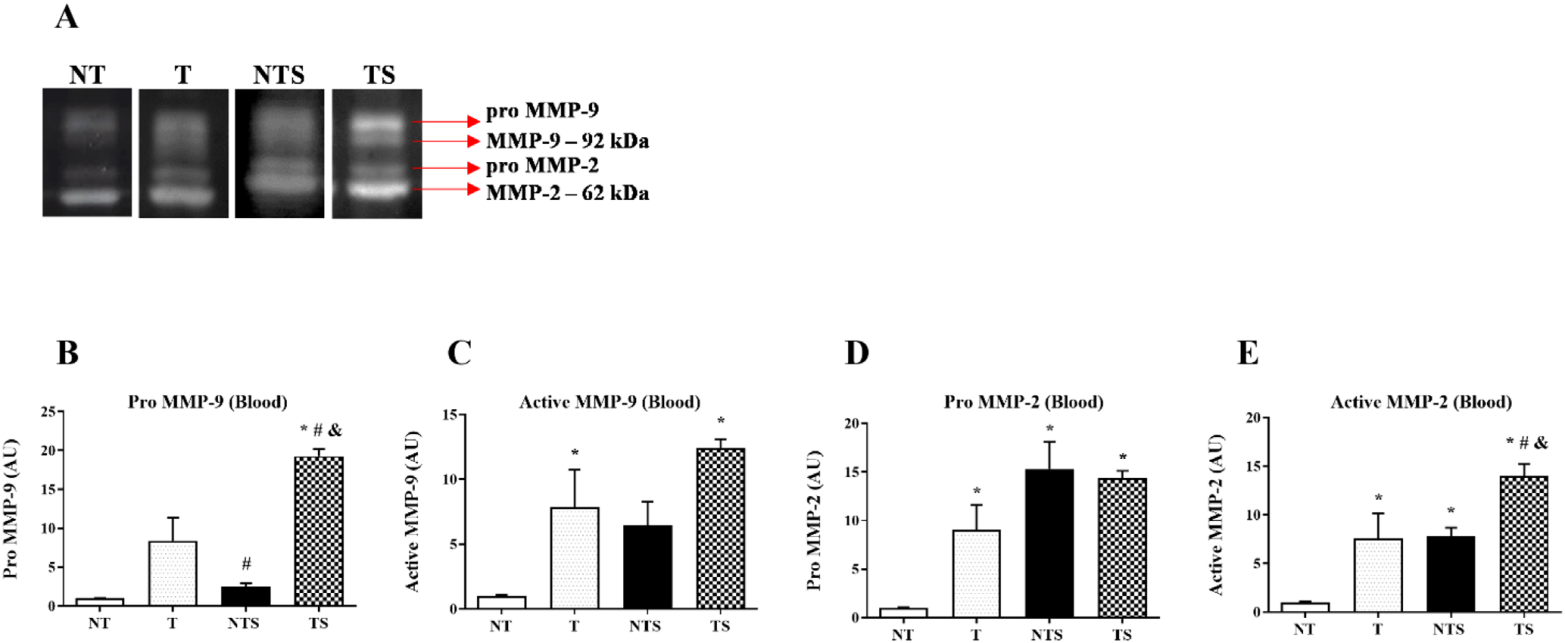
Representative zymography showing the pro and active enzymatic isoforms of circulating MMP‐9 (92 kDa), and MMP‐2 (62 kDa) (A). Densitometric analysis of circulating pro MMP‐9 (B), active MMP‐9 (C), pro MMP‐2 (D), and active MMP‐2 (E). Experimental groups: Non‐trained rats (NT); endurance‐trained rats (T); non‐trained rats submitted to chronic stress (NTS), and endurance‐trained rats subject to chronic stress (TS). Data are presented as means ± SEM (*n* = 8 per group). Two‐way ANOVA followed by Tukey post hoc test revealed a significant interaction between stress and training for pro MMP‐2 (*p* = 0.02) and pro MMP‐9 (*p* = 0.01), a main effect of stress on active MMP‐2 (*p* = 0.0003) and active MMP‐9 (*p* = 0.009), and a main effect of training on active MMP‐2 (*p* = 0.0005) and active MMP‐9 (*p* = 0.001). *vs. NT; ^#^vs. T; ^&^vs. NTS.

The circulating pro enzymatic form of MMP‐9 was substantially increased in both T (739% higher than NT; *p* = 0.001) and TS rats (1828% higher than NT; 1089% higher than T, and 1672% higher than NTS; *p* = 0.001; Figure 8A,B). The circulating active enzymatic form of MMP‐9 increased 1144% in TS group when compared to the NT group (*p* < 0.009; Figure 8A,C).

The circulating pro enzymatic form of MMP‐2 was significantly increased in T, NTS and TS groups (vs. NT; 803% higher in T than NT, 1430% higher in NTS than NT, and 1334% higher in TS than NT; *p* = 0.02; Figure 8A,D). The circulating active enzymatic form of MMP‐2 increased 675% in T rats (vs. NT; *p* = 0.0005) and 679% in NTS group (*p* = 0.0003). Furthermore, the circulating active form of MMP‐2 was substantially elevated in TS rats (1313% higher) when compared to the NT group (*p* < 0.0005), 656% higher in relation to the T group (*p* = 0.0003), and 634% higher in relation to the NTS group (*p* = 0.0005; Figure 8A,E).

**FIGURE 9:**
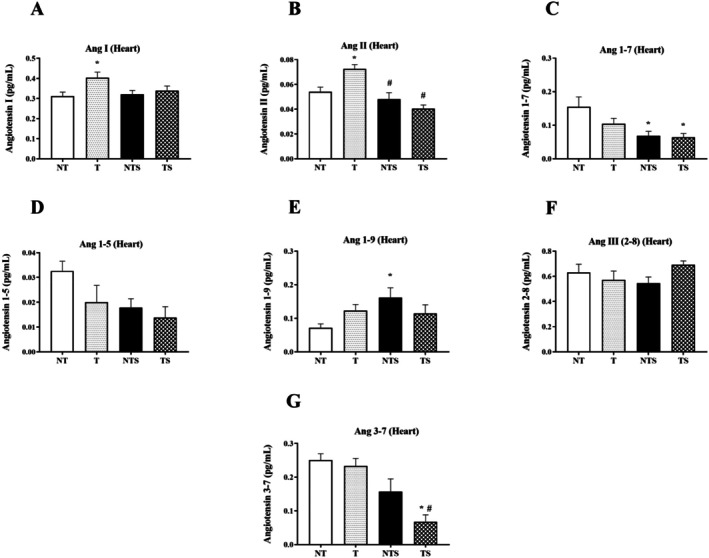
Concentrations of angiotensin peptides in left ventricle (LV) tissue, including Angiotensin I (A), Angiotensin II (B), Angiotensin 1–7 (C), Angiotensin 1–5 (D), Angiotensin 1–9 (E), Angiotensin III (2–8) (F), and Angiotensin 3–7 (G). Experimental groups: Non‐trained rats (NT); rats submitted to endurance training (T); non‐trained rats submitted to chronic stress (NTS), and rats submitted to endurance training and chronic stress (TS). Data are presented as means ± SEM (*n* = 8 per group). Two‐way ANOVA followed by Tukey post hoc test revealed significant interactions between stress and training for Ang II (*p* = 0.003) and Ang 1–9 (*p* = 0.03), a main effect of stress for Ang 1–7 (*p* = 0.002) and Ang 3–7 (*p* < 0.0001), and a main effect of training for Ang I (*p* = 0.02). *vs. NT; ^#^vs. T.

Cardiac levels of Ang I and Ang II increased in response to the endurance training (T vs. NT; Ang I, *p* = 0.02; Figure 9A), even though Ang II levels were reduced in stressed rats in comparison to T rats (*p* = 0.003; Figure 9B). Cardiac levels of Ang 1–7 were also decreased in response to the chronic stress, but when compared to NT animals (NTS and TS vs. NT, *p* = 0.002; Figure 9C). Ang 1–5 and Ang II (2–8) levels were unchanged among the groups (Figure 9D,F). While cardiac levels of Ang 1–9 increased in NTS rats (vs. NT, *p* = 0.03; Figure 9E), Ang 3–7 levels were reduced in TS when compared with NT and T rats (*p* < 0.0001; Figure 9G).

**FIGURE 10:**
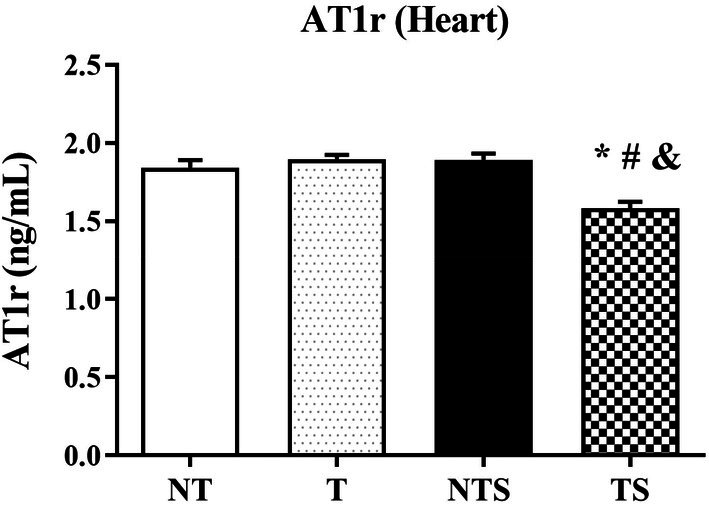
Cardiac levels of angiotensin II type I receptor (AT1r). Experimental groups: Non‐trained rats (NT); rats submitted to endurance training (T); non‐trained rats submitted to chronic stress (NTS), and rats submitted to endurance training and chronic stress (TS). Data are presented as means ± SEM (*n* = 8 per group). Two‐way ANOVA followed by Tukey post hoc test revealed a significant interaction between stress and training factors (*p* < 0.0001). *vs. NT; ^#^vs. T; ^&^vs. NTS.

Cardiac levels of AT1r were lower in TS rats than in NT, T, and NTS rats (*p* < 0.0001; Figure 10).

**FIGURE 11:**
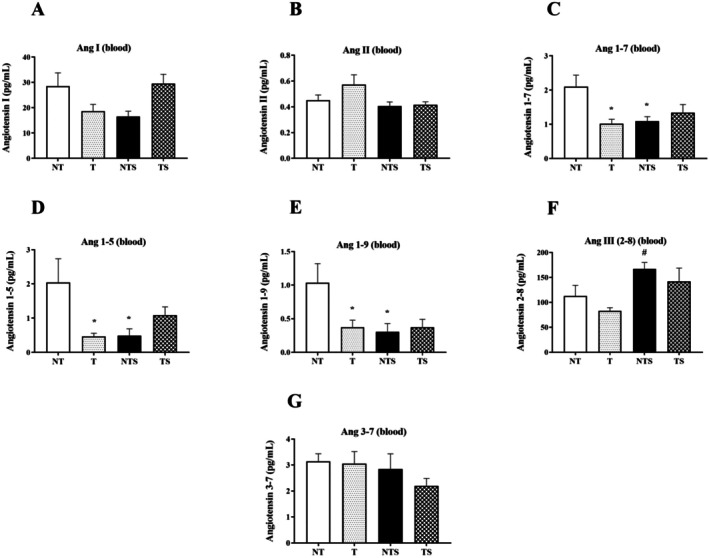
Plasma concentrations of angiotensin peptides, including Angiotensin I (Ang I) (A), Angiotensin II (Ang II) (B), Angiotensin 1–7 (Ang 1–7) (C), Angiotensin 1–5 (Ang 1–5) (D), Angiotensin 1–9 (Ang 1–9) (E), Angiotensin III (Ang III; 2–8) (F), and Angiotensin 3–7 (Ang 3–7) (G). Experimental groups: Non‐trained rats (NT), rats subjected to endurance training (T), non‐trained rats exposed to chronic stress (NTS), and rats subjected to both endurance training and chronic stress (TS). Data are presented as mean ± SEM (*n* = 8 per group). Two‐way ANOVA followed by Tukey post hoc test revealed significant interactions between stress and training for Ang 1–7 (*p* = 0.04), Ang 1–5 (*p* = 0.009), and Ang 1–9 (*p* = 0.03), as well as a main effect of stress for Ang III (2–8) (*p* = 0.003). *vs. NT; ^#^vs. T.

Circulating levels of Ang I, II, and Ang 3–7 did not change among the experimental groups (Figure 11A,B,G). T and NTS rats showed lower blood Ang 1–7, Ang 1–5, and Ang 1–9 levels than NT rats (Ang 1–7, *p* = 0.004; Ang 1–5, *p* = 0.009 and Ang 1–9, *p* = 0.03; Figure 11C–E). Circulating levels of Ang‐III (2–8) were significantly increased in response to the chronic stress when compared to trained animals (NTS vs. T, *p* = 0.003; Figure 11F).

**FIGURE 12:**
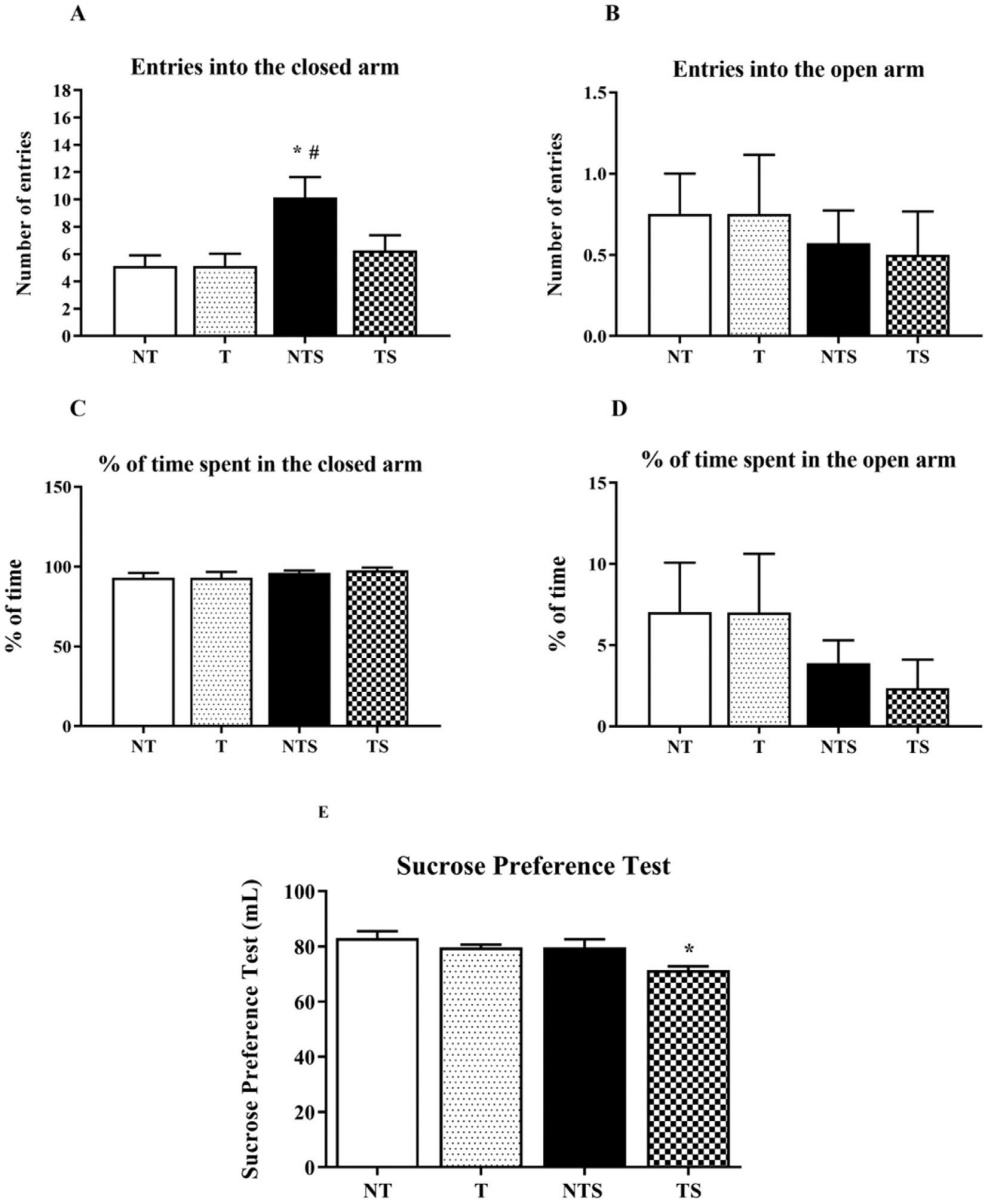
Behavioural outcomes from the Elevated Plus Maze (EPM) and Sucrose Preference Test (SPT). EPM parameters include number of entries into the closed arms (A), number of entries into the open arms (B), percentage of time spent in the closed arms (C), and percentage of time spent in the open arms (D) and results from the Sucrose Preference Test (E). Experimental groups: Non‐trained rats (NT), rats subjected to endurance training (T), non‐trained rats exposed to chronic stress (NTS), and rats subjected to both endurance training and chronic stress (TS). Data are presented as mean ± SEM (*n* = 8 per group). Two‐way ANOVA followed by Tukey post hoc test revealed a main effect of stress on the number of closed arm entries (*p* = 0.008) and sucrose preference (*p* = 0.02), and a main effect of training on sucrose preference (*p* = 0.02). *vs. NT; ^#^vs. T.

Number of entries of NTS rats into the closed arm of EPM behaviour test were significantly increased in comparison to NT and T rats (*p* = 0.008; Figure 12A). There were no differences in number of entrances in the open arm and percentage of time spent in the arms of the maze (Figure 12B–D). However, TS rats showed less preference to the sucrose when compared to NT rats (*p* = 0.02; Figure 12E).

## 4 Discussion

The present study provides new evidence regarding the associations between the stress response, endurance training adaptations, MMP activity, and angiotensin peptide levels. Variations in corticosterone levels may represent an initial triggering factor associated with downstream intracellular signalling pathways related to changes in MMP activity and angiotensin peptide levels. Lower cardiac levels of Ang 3–7 observed in stressed rats might be associated with a protective adaptation induced by aerobic training during chronic stress exposure, although increased antifibrotic MMP‐2 activity was observed only in the circulation, and not in cardiac tissue. In addition, chronic stress was associated with alterations in MMP activity and RAS peptide profiles, as evidenced by increased cardiac MMP‐2 activity and elevated circulating Ang 1–9 levels, accompanied by reduced cardiac and circulating levels of Ang 1–7. These responses were also associated with reduced circulating levels of Ang 1–9 and Ang 1–5 together with increased Ang III (2–8).

Although mental stress has been associated with abdominal obesity due to altered food intake behaviour [41, 42], experimental studies have consistently shown reduced body weight gain in stressed animals [9, 43, 44, 45]. In this study, TS rats displayed reduced weight gain compared to T rats during the stress protocol (weeks 4–6; Figure 2), indicating its potential adverse impact on body composition. Despite these differences during the stress period, no significant differences in body weight were observed among the experimental groups after 8 weeks. Notably, corticosterone, a key hormone regulating the stress response in rodents [46], was significantly elevated in NTS rats compared to NT and T rats (Figure 3A). On the other hand, aerobic endurance training mitigated the stress‐induced elevation of the circulating corticosterone levels (TS vs. NTS), underscoring its protective effect against stress‐related responses. As the weight management and corticosterone were significantly affected by the CMS protocol and endurance training, it is reasonable to expect ensuing physiological and behavioural responses. In fact, chronic stress markedly increased circulating ALT levels exclusively in trained rats (TS vs. NT, T, and NTS; Figure 4B) and caused a moderate increase in AST levels (TS vs. T; Figure 4C). ALT and AST are widely used markers of liver function, although AST can also be released from cardiac and skeletal muscle tissues. While ALT is involved in gluconeogenesis and amino acid metabolism [47], AST is associated with various tissue injuries, including those of the myocardium [48]. Our findings align with previous studies showing that chronic mild unpredictable stress can elevate ALT and AST levels [49, 50] even though some reports have found no changes in these markers under similar conditions [51]. The increased ALT and AST levels in TS rats suggest systemic metabolic disruptions and a potential cardiovascular impact of stress during physical training. These observations are further supported by evidence linking the AST/ALT ratio with arterial stiffness and pulse wave velocity [52], reinforcing the idea that liver injury markers might reflect stress‐related cardiocirculatory disturbances and adaptations to exercise.

Because depressed mood can cause psychomotor inhibition [5, 53], we expect that chronic stress would reduce the locomotor activity and, therefore, impact negatively the endurance performance. Although both trained groups (TS and T) showed better endurance performance at test 2 (which corresponds to the end of the week #4 of the experimental period) than the non‐trained rats, the intriguing aspect was the fact that the endurance performance was not decreased in TS rats at test #3 (corresponding to the end of the week #8 of the experimental period) when compared to the other experimental groups. It is possible that a preventive effect whereby aerobic endurance training might be protecting against the stress‐induced impairments on treadmill performance. In fact, strain differences were shown to impact the locomotor activity [54] as we have demonstrated that stress mitigated the endurance performance in trained rats in a previous study, using the Sprague Dawley strain [9]. In contrast to our previous study, Wistar rats submitted to endurance training and chronic stress (TS group) showed improved endurance performance (Figure 3A), which could be associated with the upregulation of enzymes related to the aerobic energy metabolism [47], including CS and CK, in active muscles of TS rats. In fact, the better performance observed in TS rats was accompanied by a tendency of higher CS activity (a marker of mitochondrial biogenesis) in soleus (SOL), but not in gastrocnemius (Gastroc) muscle (Figure 3B,C). On the other hand, the combination of endurance training and chronic stress attenuated the stress‐triggered increase in the circulating CK (biomarker of tissue damage—skeletal muscle, heart and brain) levels (Figure 3D,E). Although some controversies exist regarding whether CK activity is the best biomarker of muscle damage induced by endurance physical activities [55, 56, 57, 58, 59], our findings suggest for the first time an increase in the circulating CK activity induced by chronic stress, which could indicate a target for stress‐related cardiovascular events, supported by previous studies demonstrating that either acute or chronic stress elevated plasma CK in rats [60, 61] and after a single bout of stressful, prolonged, intense exercise [62]. In fact, CK has been associated with hypertension [63] while CK‐MB is found predominantly in cardiac muscle and is used as a biomarker of myocardial damage and kidney diseases [64]. Noteworthy, we observed that aerobic endurance training abrogated the stress‐induced high circulating levels of CK and CK‐MB, suggesting a protective role against cardiorenal damage (Figure 3D–F).

Considering that the combination of endurance training and chronic stress can provoke significant changes in the circulating biomarkers related to skeletal muscle and myocardial injury, it is tempting to assume that autonomic and hemodynamic regulation could be affected. Accordingly, the left ventricle‐to‐body weight ratio (LV/BW) was reduced in TS rats (vs. T and NTS groups; Figure 5A) while the left ventricle to‐tibia length ratio (LV/tibia, an index of LV hypertrophy) increased in response to endurance training. Conversely, stress reduced the LV/tibia ratio when compared to the trained rats (Figure 5B). This cardiac phenotype modification could indicate that the physiological hypertrophic response induced by the endurance exercise training is abrogated by chronic stress. Since chronic exercise training induces physiological hypertrophic stimuli and is associated with preserved or enhanced cardiac function [65], we hypothesized that stress could negatively influence the autonomic regulation and systemic hemodynamics. Neither aerobic endurance training nor chronic stress changed the SBP and MBP (Figure 5C,D). However, endurance training significantly reduced HR (Figure 5E). Whereas chronic stress abrogated the training‐induced bradycardia. The reduction in HR induced by aerobic training has been associated with enhanced cardiac parasympathetic regulation [66, 67]. Therefore, our findings suggested that chronic stress counteracts the beneficial effects of endurance training on cardiac balance likely through alterations in intrinsic electrophysiological properties, particularly sinoatrial node pacemaker activity [66, 68].

Whereas some studies have reported that psychosocial stress increases HR in mice, as assessed by echocardiography on the final day of the stress protocol [69], others have found no effect of chronic variable stress on HR [70] which is consistent with our observations. Importantly, Agrimi et al. [69] also demonstrated preserved systolic and diastolic function despite chronic stress exposure and increased HR, suggesting that alterations in cardiovascular parameters do not necessarily indicate overt cardiac dysfunction. Similarly, Bruder‐Nascimento et al. [71] reported that chronic stress induced cardiac hypertrophy without impairing baseline left ventricular or myocardial function in rats, reinforcing the concept that structural remodelling may occur independently of measurable functional decline. Collectively, these studies support the interpretation that stress‐induced alterations in cardiovascular parameters may reflect early maladaptive changes in cardiac remodelling and cardiovascular regulation rather than definitive evidence of impaired cardiac function. Therefore, our findings should be interpreted as evidence of stress‐related cardiac remodelling and altered cardiovascular regulatory pathways, which may precede overt systolic or diastolic function. Although endurance training alone did not significantly affect cardiomyocyte width, the combination of endurance training and chronic stress was associated with increased cardiomyocyte width (Figure 6), suggesting structural remodelling in response to the combined exposure to endurance training and chronic stress. Considering the complex helical organization of myocardial fibres throughout the ventricular wall, the present measurements should be interpreted as an approximate morphometric index rather than a definitive assessment of cardiomyocyte hypertrophy, since variations in sectioning angle and fibre orientation may directly influence the apparent dimensions of cardiomyocytes in histological analyses.

To further explore the molecular mechanisms whereby chronic stress exerts detrimental effects on those cardiovascular events, we investigated the activity of the MMPs and angiotensin levels in heart and circulation. Proteases represent one of the most abundant functional classes of proteins and are categorized into five major catalytic groups (metalloproteinases, serine, cysteine, threonine, and aspartic proteinases), with metalloproteinases constituting the largest group [72]. MMPs are zinc‐dependent endopeptidases involved in the degradation and remodelling of ECM components, including denatured fibrillar collagen and basement membrane proteins (collagen type 4, fibronectin, and laminin) [20]. Moreover, MMP‐2 and MMP‐9 exhibit proteolytic activity against elastin and proteoglycans [20] and play a pivotal role in the cytokine‐mediated inflammatory processes [73]. MMP‐2 and MMP‐9 are distinguished from other MMPs by the presence of three fibronectin type II motif repeats in the catalytic domain [74] and can be activated by non‐proteolytic mechanisms, such as peroxynitrite and glutathione, potential effectors of oxidative stress [75, 76]. ProMMPs, the latent (inactive) state of MMPs, are secreted as inactive zymogens and remain enzymatically quiescent until the propeptide domain is cleaved by proteinases [20, 77]. The activation of MMP‐2 by oxidative stress enhances glycogen synthase kinase‐3β activity, which may contribute to cardiac damage [78]. The main activation of proMMP‐2 is not triggered by general proteinases but is mediated by membrane‐type MMPs (MT‐MMP) on the cell surface [77]. Following the initial proteolytic cleavage (and release of the propeptide sequence) by serine proteases, the intermediate form of MMPs is partially activated by interacting with their endogenous tissue inhibitor of MMPs (TIMPs) [77, 79] and MT1‐MMP [80]. When fully activated, MMP‐2 degrades collagens, elastin [81], and cardiac contractile proteins [82], while its intracellular activity contributes to the myocardial ischemia–reperfusion (IR) injury [74]. Furthermore, MMP‐2 can induce contractile dysfunction by targeting numerous intracellular proteins in cardiac myocytes, including troponin I, α‐actinin, myosin light chain‐1, and junctophilin‐2 [78]. Therefore, MMP‐2 shows promise as a tissue marker to evaluate the potential effects of chronic stress on cardiac tissue. Whereas the latent state pro‐MMP‐2 increased with endurance training (Figure 7B), a tendency for increased intermediate form and a marked increase in the cardiac MMP‐2 activity (active state) were observed in stressed rats (Figure 7D). Thus, it seems plausible to hypothesize a strong association between cardiac MMP‐2 activity and plasma corticosterone in the face of potentially stressful challenges. On the other hand, aerobic endurance training substantially reduced not only the intermediate form but also the active form of the cardiac MMP‐2, providing strong evidence for the cardio‐protective effects of aerobic physical training against the stress‐related cardiovascular complications, as observed by others [15].

In addition, both aerobic training and chronic stress elevated the pro and active enzymatic forms of MMP‐2 in the blood circulation, whereas aerobic endurance training greatly increased the circulating MMP‐2 in stressed rats (Figure 8C,D). The circulating MMPs levels might reflect the secretory activity from multiple tissues, including lung, stomach, colon, small intestine, bone marrow, lymph nodes, adrenals, spleen, kidneys, adipose tissue, prostate, skin, bladder, testes, and central nervous system [83] and, therefore, represent important sources for circulating MMP‐2 and ‐9. The elevated levels of blood MMP‐2 observed in TS rats could be related not only to the higher levels of circulating ALT (Figure 4B) but also to the cardiomyocyte width (Figure 6A,B). In this sense, MMP‐9 also has been shown to serve as a proximal biomarker for cardiac remodelling and tightly linked to the inflammatory response [84, 85]. MMP‐9 is involved in the proteolytic activation of other important biologically active proteins/peptides, such as transforming growth factor TGF‐ß, cell surface receptors, and cell adhesion molecules (CAMs) [86, 87, 88], while it has been shown to be a critical regulator of ECM remodelling, hippocampal development, synaptic plasticity, neuroinflammation, and brain‐damaging injuries [72, 87, 89]. Although the circulating levels of the active isoform of MMP‐9 was not affected by chronic stress (Figure 8C), exercise training was shown to induce a marked increase in both the pro and active MMP‐9 levels in the circulatory system (Figure 8B), partially consistent with previous studies [90, 91]. Studies on gelatinases in blood showed an early release of MMP‐9 following acute exercise of sufficient intensity, whereas MMP‐2 levels were more contrasting [92]. In this sense, angiotensin II (Ang II) peptide induces an early increase in aortic MT1‐MMP expression with a subsequent increase in MMP‐2 and MMP‐9 activity [93], suggesting an intricate complex interplay among MMP‐2 activation and the cardiovascular responses in the context of chronic stress and aerobic endurance training. Furthermore, as endurance training activates MMPs in skeletal muscle [94], we suggest that the higher levels of the circulating MMPs are most likely due to the skeletal muscle activity and the following MMP release into the bloodstream. In conclusion, evidence from experimental and animal studies on the role of aerobic endurance training on plasma MMPs level remains inconsistent [13, 95, 96]. Additionally, increases in the expression of pro‐ and/or active isoforms of MMP‐9 have been observed in the hippocampus, while some studies have also reported MMP‐9 activation in the cerebral cortex [88]. MMP‐9 activation has also been observed in specific brain structures following learning in some behavioural tasks, such as the Morris water maze, inhibitory avoidance, contextual fear conditioning, object exploration, and response habituation [88], which provides new compelling information on the MMP‐9‐mediated effects of endurance training on brain function and cognitive behaviours.

Our findings may ultimately provide a better understanding of some cardiometabolic disorders and mental‐related diseases by using MMPs and liver transaminases (ALT and AST) as stress biomarkers [97, 98]. Given that the ECM participates in neuroplasticity and has been linked to cognition and emotional regulation, ECM components may be candidate therapeutic targets for stress‐induced neuropsychiatric disease [99].

Considering that Ang II, the main effector peptide of the RAS, plays a determinant role in the cardiovascular remodelling [100, 101], we explored the effects of endurance training combined with chronic stress on the angiotensin profile. Although several studies have characterized the RAS in cardiovascular diseases [102, 103], no study has explored whether aerobic endurance training could protect the cardiac damage attributable to chronic stress by regulating circulating and tissue angiotensin levels. Our findings revealed that chronic stress, associated or not with aerobic endurance training, mostly affected the counter‐regulatory axis of the RAS. Emerging evidence supports that the peptides from the counter‐regulatory peptides, as part of the opposite effects to classic RAS, may also elicit anti‐inflammatory and antifibrotic effects [104]. Lower levels of cardiac Ang 1–7 (Figure 9C) and increased levels of Ang 1–9 (Figure 9E) were observed in response to chronic stress. Ang 1–7 is a heptapeptide generated from Ang II by angiotensin converting enzyme 2 (ACE2) and Ang I by peptidases [100]. It has been postulated that Ang 1–7 and its metabolite Ang 1–5 [105] can exert cardioprotective [106] and renoprotective [107] effects by counterbalancing many Ang II actions [108]. In fact, Ang 1–7 can reduce the elevated heart rate associated with emotional stress and decrease anxiety [109]. In this sense, a study demonstrated anxiolytic‐like effects in response to the central administration of Ang 1–7 [110]. Thus, our findings suggest that chronic stress could have evoked cardiac disturbances by lowering Ang 1–7 levels in heart (Figure 9C) and circulatory system (Figure 10C), which abrogates the protective effects of aerobic endurance training on lowering heart rate (HR) (Figure 5E). Furthermore, as stress affects Ang 1–7 levels, it would be possible to observe changes in anxiety or depressive‐like behaviours of stressed rats, as documented by others [109, 110]. The reduced circulating levels of Ang 1–7 in rats submitted to stress supports this conjecture, even though aerobic endurance training also decreased the circulating levels of Ang 1–7 in non‐stressed rats (Figure 11C). In this way, the increased cardiac Ang 1–9 levels (Figure 9E) in response to chronic stress can contribute to the deleterious effects on heart. Ang 1–9 is produced from Ang I and exerts Ang II‐like prothrombotic effect in the circulatory system of rats [111] while stimulating the atrial natriuretic peptide (ANP) secretion [105] and bradykinin in endothelium, with antihypertrophic and antifibrotic actions in the heart [112, 113, 114] as well as to attenuate cardiac dysfunction in diabetic rats [115]. However, the cardiovascular improvements induced by Ang 1–9 appear to be mediated by the AT2 receptor, but not by the angiotensin‐(1–7)/Mas receptor axis, suggesting the RAS receptors exert a crucial role in the cardiovascular outcomes. In this sense, the combination of aerobic endurance training and chronic stress led to a marked reduction in the cardiac AT1 receptor (Figure 10). In our observations, Ang III (2–8) (Figure 9F) and Ang IV (3–8) could be participating in the cardiac AT1r downregulation (Figure 10). Cardiac Ang III (2–8) showed a tendency to increase in TS rats, which could favour a negative feedback loop to the AT1r downregulation. In the context of numerous pathologies, reactive oxygen species (ROS) generated by vascular NADPH oxidases also activate the AT1 receptor [116, 117]. Remarkably, we have reported that the levels of thiobarbituric acid reactive substances (TBARs), an index of lipid peroxidation, were significantly elevated in hearts of rats submitted to aerobic endurance training and chronic stress [9], which might be contributing to the cardiac AT1r downregulation.

Higher cardiac Ang 1–9 levels in NTS rats could represent a contradictory result in view of the adverse effects induced by chronic stress on cardiac tissue. However, circulating levels of Ang 1–9 were reduced in rats submitted to chronic stress, while aerobic endurance training prevented this response in stressed rats (Figure 11E). In fact, not only the circulating levels of Ang 1–9 but also the Ang 1–7 and its metabolite Ang 1–5 [105] were reduced in T and NTS rats (Figure 11D). Similarly to those peptides, Ang 1–5 offers cardioprotection against ischemia–reperfusion (I/R) injury [108] and stimulates ANP secretion via Mas receptor [105]. Therefore, we suggest that the main protective effects of aerobic endurance training are not mediated by the circulatory and intracellular levels of Ang 1–9, Ang 1–7, and Ang 1–5 peptides, whereas chronic stress represents an aversive stimulus that dramatically influences the levels of the peptides related to the counter‐regulatory RAS peptides.

Noteworthy, cardiac Ang 3–7 levels decreased when aerobic endurance training was combined with chronic stress (Figure 9G), with a tendency towards lower levels in the circulatory system (Figure 11G). Ang 3–7 is a fragment of the RAS that can be derived from Ang II or Ang 1–7 [118] and exerts pressor effects at the rostral ventrolateral medulla (RVLM) [118]. Although Ang 3–7 was recently shown to alleviate cardiac remodelling in neonatal rats [119], the mechanism underpinning the cardioprotective effects of aerobic endurance could be related to the reduced sympathetic neural activity along with lower pressor responses, dictated by the lower levels of cardiac Ang 3–7. Moreover, Ang 3–7 stimulates behavioural activity of rats similar to Ang II [120] and plays a role in psychoactive properties [121], suggesting that the combination of aerobic training and chronic stress exerts potential effects on behavioural responses with pronounced influence of the counter‐regulatory RAS peptides. In blood, only Ang III (2–8) was increased in response to chronic stress (Figure 11F). In fact, Ang III (2–8) has similar functions to Ang II [100], such as aldosterone secretion [122], vasopressin release [123], DNA and collagen synthesis in cardiac fibroblasts, and water intake [124]. Park et al. [125] showed cardioprotective effects of Ang III against I/R injury by activating antioxidant and antiapoptotic enzymes via AT2R and KATP channels, but this study was conducted in perfused hearts and may evoke divergent implications. The mechanism(s) underpinning the counter‐regulatory RAS peptides remain unclear in the setting of physical training associated or not with chronic stress, although the counter‐regulatory RAS peptides appear to be strictly regulated by stressful conditions.

Ang II is an important factor related to neurodevelopment and regulation of the stress response [17]. Given that chronic stress disrupted the balance of counter‐regulatory peptides within the RAS, it is reasonable to infer that behavioural impairments would be evident in stressed rats. In contrast, aerobic endurance training may prevent the stress‐like behaviours, as previously suggested by others [10]. Several studies have consistently show the negative effects of stress on behaviours patterns of experimental models [40, 126, 127, 128]. Data on locomotion, anxiety, exploration, and other behaviours in CMS‐exposed rodents often demonstrate paradoxical and conflicting behavioural changes [5]. In the present study, non‐trained rats exposed to chronic stress (NTS) exhibited an increased number of entries into the closed arms of the EPM (Figure 12A). Since closed arm entries are more closely related to general exploratory activity, rather than anxiety‐like behaviour per se [129], these findings suggest that CMS induced alterations in behavioural reactivity, which may reflect changes in both locomotor and emotional components. Importantly, no significant differences were observed in either the number of entries into the open arms or the time spent in the open arms. The lack of significant changes in classical anxiety‐related parameters (e.g., open arm exploration) reinforces that the observed behavioural changes are not exclusively anxiety‐driven. Importantly, these behavioural changes were observed in parallel with increased corticosterone levels and reduced sucrose preference, as discussed in detail below, indicating that, despite the absence of differences in classical anxiety‐related parameters in the EPM, the CMS protocol effectively induced a stress‐related phenotype with depressive‐like features. Therefore, the EPM findings should be interpreted cautiously, as alterations in exploratory or locomotor activity may have contributed to the behavioural outcomes observed in the present study. Aerobic endurance training did not elicit improvements in the rats behaviour, even though exercise training (aerobic and resistance training) has been shown to improve depression‐like behaviours in the stressed rats [130, 131]. Our findings did not reveal improvements on trained rats submitted to chronic stressed rats, which could be explained by the differences between exercise protocols. For example, Liu and colleagues used a 30 min/day endurance exercise protocol on a motor‐driven treadmill, at zero degree slope with a progressive speed daily, 5 days per week for 4 weeks [47], whilst we have used an endurance training protocol of 60 min duration, 5 days/week, for 8 weeks [9].

Anhedonia, a core feature of depressive‐like behaviour, is characterized by a diminished capacity to experience pleasure, often accompanied by a persistently low mood [5]. Sucrose preference test (SPT) is the most frequently employed method used for assessing anhedonia in rodents [5]. We observed that only TS groups exhibited anhedonia (Figure 12E), which might be strongly associated with reduced sweet taste perceptions following mental stress [132, 133]. Variabilities in sucrose test results can be explained by inter‐strain and inter‐batch differences in experimental animals, as well as the high sensitivity of drinking behaviour and sucrose intake to internal and external conditions [4, 127]. Furthermore, a plenty of factors have been shown to impact the outcomes of the SPT test, as recently reviewed by Strekalova and colleagues (2022). Those factors include the effects of stressors present during the sucrose test or the lasting action of previously applied stressors on consummatory behaviour, sugar concentration, diet and water deprivation, neophobia, social status of rodents, sensitization to reward experiences during repeated or prolonged exposure to palatable solutions, fluctuations in calorie and water intake due to differences in body mass and altered equilibrium of sympathetic/parasympathetic regulation, strain and inter‐individual variabilities in liquid and sucrose intake, circadian rhythms, and inter‐batch variability [5]. In addition to that, we suggest that the high volume (frequency × duration) of the aerobic endurance training (main effect of training, *p* = 0.02) could represent a potential factor contributing to the anhedonia in TS rats as well as the ALT levels could lead to the anhedonia (Figure 12).

**FIGURE 13:**
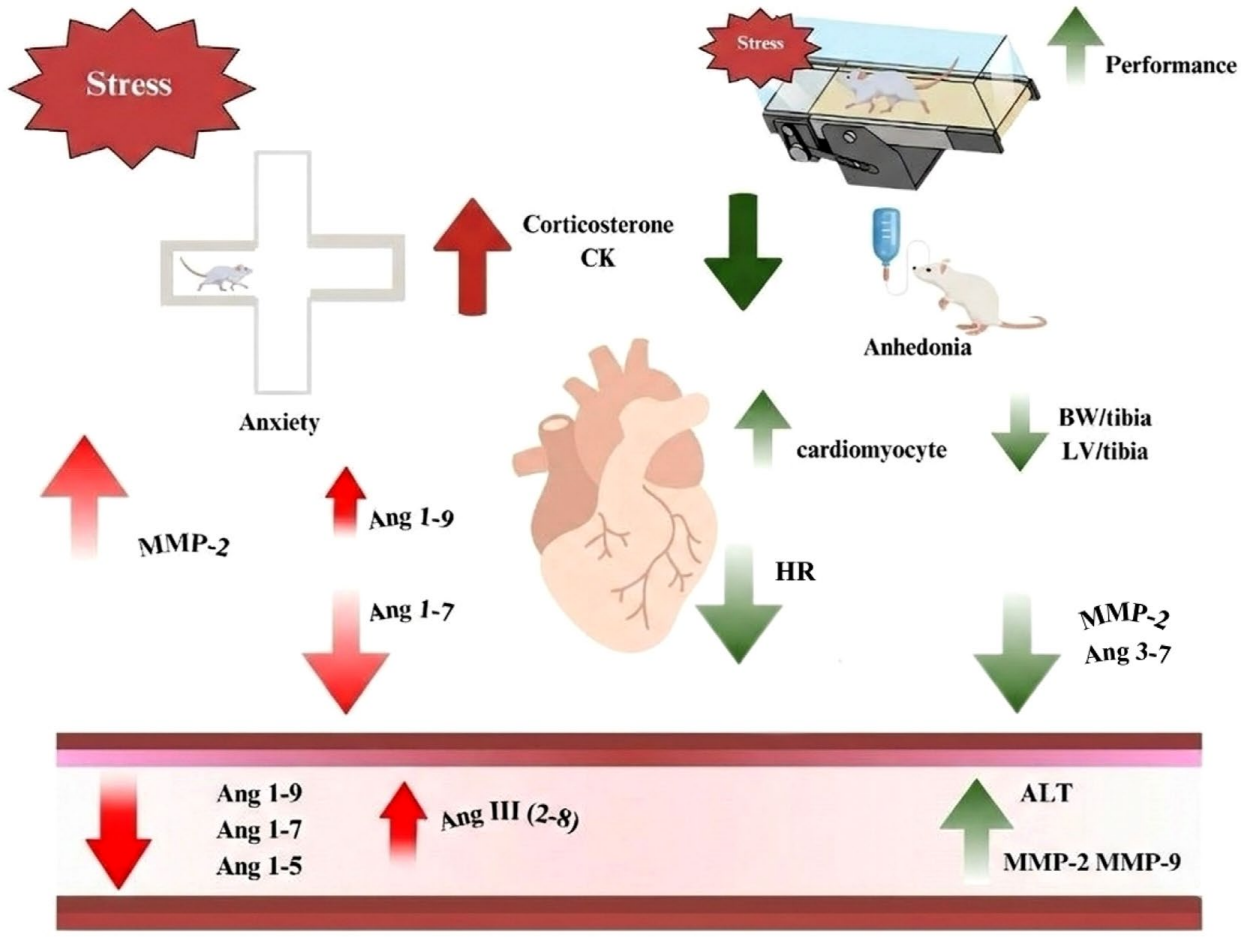
Schematic illustration depicting the combined effects of aerobic endurance training and chronic stress (green arrows), as well as the effects of chronic stress alone (red arrows). This diagram highlights the complex interplay between counter‐regulatory angiotensin signalling and matrix metalloproteinase (MMP) activation. HR, Heart rate. Illustration created with www.canva.com.

This study provides additional experimental evidence linking endurance training and chronic stress to alterations in MMP activity and angiotensin peptides, further supporting and extending current understanding of their integrated physiological responses. Our findings suggest that corticosterone may act as a potential upstream modulator associated with changes in intracellular signalling pathways involving MMPs activation and molecules of the RAS. Endurance training was associated with cardioprotection adaptations against the stress response, including reduced levels of Ang 3–7, a derivative of Ang II, together with alterations in MMP activity in trained rats exposed to CMS. These findings may reflect an association between changes in the counter‐regulatory RAS peptides and MMPs‐related extracellular matrix remodelling, although direct mechanistic interactions were not evaluated in the present study. Chronic stress, in turn, was associated with alterations in cardiac remodelling and cardiovascular regulatory parameters, along with changes in both MMP and RAS profiles, characterized by increased cardiac MMP‐2 activity and Ang 1–9 levels, together with reduced cardiac and circulating levels of the cardioprotective peptide Ang 1–7. These findings suggest a shift in cardiac structural and regulatory dynamics. Additionally, stress was associated with decreased circulating Ang 1–9 and Ang 1–5, an Ang 1–7 metabolite, as well as increased Ang III (2–8), a derivative of Ang II. Although chronic stress negatively impacted these pathways, aerobic endurance training was associated with partial attenuation of stress‐related alterations in MMP and RAS signalling. The main responses to the aerobic endurance training and chronic stress were summarized in Figure 13.

### 4.1 Limitations

Some limitations of the present study should be acknowledged. Only one sex was studied, which limits the generalizability of the findings and precludes the assessment of sex‐specific responses. An important limitation of this study is the absence of direct assessment of cardiac function (e.g., echocardiography). Despite recognized methodological limitations, the tail‐cuff technique remains widely accepted and commonly used in experimental studies for comparative analyses across groups [134, 135]. In addition, cardiomyocyte hypertrophy was evaluated using width measurements, a two‐dimensional morphometric index that, although widely used, does not fully capture the three‐dimensional structure of cardiomyocytes. Another potential limitation of the present study is the absence of an independent assessment of locomotor activity, such as the open field test. Although the EPM is widely used to assess anxiety‐like behaviour, it is recognized that some of its parameters, particularly total arm entries and closed arm entries, are commonly used as indices of general motor activity. In addition, the use of relative measures, including the percentage of open arm entries or time, may partially account for differences in locomotion across groups [129]. Nevertheless, these approaches do not fully dissociate locomotor activity from anxiety‐like behaviour. Finally, this study is primarily associative in nature, limiting causal interpretation of the interactions between endurance training, chronic stress, MMP activity, and RAS peptides. Despite these limitations, the findings provide relevant insights into the integrated physiological responses to endurance training and chronic stress, while highlighting areas for future mechanistic investigation.

## Author Contributions

**Fabiana Gomes Ferreira:** conceptualization, investigation, methodology, validation, writing – review and editing, formal analysis, data curation. **Vinicius Guzzoni:** conceptualization, investigation, writing – original draft, methodology, validation, writing – review and editing, visualization, formal analysis, data curation. **Lívia Bruni de Souza:** investigation, methodology, validation, writing – original draft, data curation, formal analysis. **Rebecca Salomão:** investigation, validation, methodology, data curation. **Elia Garcia Caldini:** investigation, methodology, validation, formal analysis, data curation. **Nicholas Kesler:** writing – review and editing, validation, data curation, formal analysis. **Fernanda Klein Marcondes:** conceptualization, investigation, writing – original draft, writing – review and editing, formal analysis, data curation, methodology, validation, visualization. **Rodrigo Yokota:** methodology, writing – review and editing, writing – original draft, data curation, investigation, formal analysis. **Nilsa Regina Damaceno‐Rodrigues:** methodology, validation, investigation, data curation, formal analysis. **Rita de Cássia Marqueti:** writing – review and editing, methodology, formal analysis, data curation, visualization, investigation. **Steven T. Haller:** writing – review and editing, validation, data curation, visualization, investigation. **David J. Kennedy:** writing – review and editing, validation, visualization, data curation, investigation. **Lauren G. Koch:** investigation, validation, visualization, writing – review and editing, data curation. **Dulce Elena Casarini:** conceptualization, investigation, funding acquisition, writing – original draft, writing – review and editing, formal analysis, data curation, supervision, resources, validation, methodology, visualization, project administration. **Tatiana Sousa Cunha:** conceptualization, investigation, funding acquisition, supervision, resources, data curation, formal analysis, project administration, visualization, validation, methodology, writing – review and editing.

## Funding

This work was supported by the Coordenação de Aperfeiçoamento de Pessoal de Nível Superior (CAPES)—Postgraduate Program in Translational Medicine (UNIFESP), Conselho Nacional de Desenvolvimento Cientifico e Tecnologico (CNPq), and Fundação de Amparo à Pesquisa do Estado de São Paulo [FAPESP Processes 2021/06090‐8 and 2013/00798‐2].

## Conflicts of Interest

The authors declare no conflicts of interest.

## Data Availability

The data that support the findings of this study are available on request from the corresponding author. The data are not publicly available due to privacy or ethical restrictions.
